# Modern Developing Directions in the Dihydroquercetin Study

**DOI:** 10.3390/molecules30214187

**Published:** 2025-10-26

**Authors:** Svetlana Yu. Filippovich, Elena P. Isakova, Galina P. Bachurina, Yulia I. Deryabina

**Affiliations:** A.N. Bach Institute of Biochemistry, Research Center of Biotechnology of the Russian Academy of Sciences, Leninsky Ave. 33/2, 119071 Moscow, Russia; syf@inbi.ras.ru (S.Y.F.); elen_iss@mail.ru (E.P.I.); galainbi@yandex.ru (G.P.B.)

**Keywords:** dihydroquercetin, bioavailability, solubility, nanotechnology, antibacterial, antiviral, antiage

## Abstract

Dihydroquercetin (taxifolin) is a natural bioflavonoid with diverse biological activities, including antioxidant, anti-inflammatory, antibacterial, antiviral, and geroprotective effects. The review summarizes current advances in the research of dihydroquercetin with a focus on its structural features, physicochemical properties, and biological functions. Particular attention is drawn to its low solubility and limited bioavailability, which have stimulated the development of nanotechnology-based delivery systems. In the paper, the systematization of the systems, namely nanoparticles, liposomes, hydrogels, nanofibers, nanoenzymes, and crystalline complexes, is presented. Moreover, some specific features of the antibacterial and antiviral action of the compound are described. Recent findings on the molecular mechanisms of action, including regulation of oxidative stress and cellular signaling pathways, highlight the therapeutic promise of dihydroquercetin. Taken together, the data support its potential as a valuable compound for pharmaceutical and biomedical applications, though further comprehensive investigations are required.

## 1. Introduction

**Figure 1:**
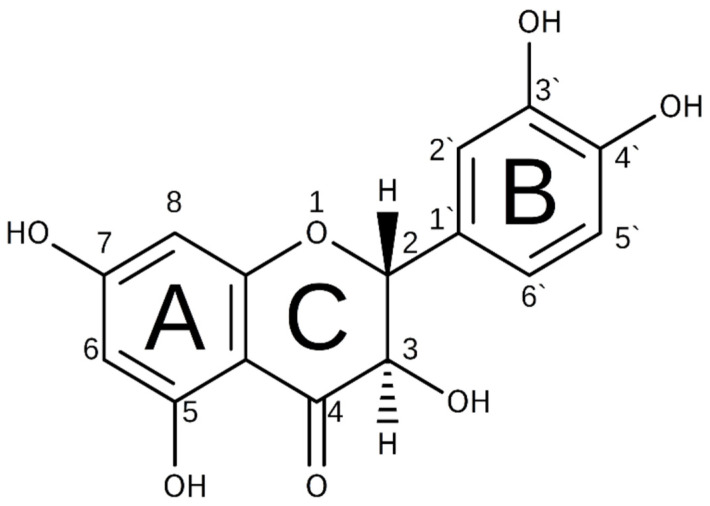
Structural formula of DHQ.

Dihydroquercetin (DHQ), also known as taxifolin, belongs to the subclass of flavanonols in the flavonoid family. Figure 1 shows its formula of 2-(3,4-dihydroxyphenyl)-3,5,7-trihydroxy-2,3-dihydro-4H-1-benzopyran-4-one. A wide spectrum of biological effects of the polyphenol includes antioxidant, antibacterial, antiviral, anti-inflammatory, and anti-apoptotic activities. In addition, the compound is capable of functioning as a hepatoprotective, antidiabetic, cardioprotective, anticancer, and brain protection agent [1,2,3,4,5,6,7]. Being a powerful natural antioxidant and acceptor of free radicals, it exhibits activities that can improve human health, and its beneficial disease-preventing properties are the subject of many studies. This polyphenol is often used as an active dietary additive and is included in many medicinal herbal mixtures. In Russia and the European Union, it is applied as an active pharmacological agent with the recommended oral daily dosage of 25 and 100 mg, respectively [8,9]. It was revealed that sublingual application of the commercially available DHQ drug is more efficient compared to the peroral one. Over 340 biologically active substances and food supplements enriched with DHQ are registered in the Eurasian Economic Union [8]. Dietary DHQ additives may be useful not only for humans but also for agricultural applications, particularly in commercial husbandry, poultry, and apiculture [10,11]. DHQ is able to influence the availability of nutrients in the diet and the growth of animals by changing their antioxidant properties.

The main limitations and gaps in the use of DHQ are poor bioavailability, low stability, and rapid metabolism, which reduce its therapeutic efficacy. Furthermore, the stereoisomers of this compound have been studied insufficiently. Recently, due to the accumulation of new nanotechnological approaches to increasing the bioavailability of DHQ, there has been a need to systematize all these results, and this became one of the goals of the presented review. Furthermore, the enhanced interest in the flavanonol is associated with the search for safe natural compounds that could successfully compete with chemically synthesized antiviral and antibacterial drugs. Moreover, due to the pleiotropic effects of DHQ on various targets in the human body, this polyphenol has promising potential for use as an anti-aging agent. Thus, the present review is focused on the current achievements in the DHQ study, namely improvement of its bioavailability and its role in combating bacteria, viruses, and aging.

The narrative review is based on original papers devoted to modern developing directions in the DHQ study. Only papers written in English were selected. The manuscript search was conducted using ScienceDirect, PubMed, and Google Scholar databases, including articles up to 1 July 2025. The keywords “dihydroquercetin” and ‘taxifolin” in combination with the terms “bioavailability”, “bioaccessability”, “nanotechnology”, “solubility”, “antibacterial”, “antiviral”, and “antiage” were used. Only the manuscripts strictly related to the review topic were selected.

## 2. General Properties of DHQ

### 2.1. Sources

DHQ was found in different gymnosperms like *Larix sibirica* (Siberian larch), *Larix gmelini* (Dahurian larch), *Larix decidua* (European larch), *Larix kaempferi* (Japanese larch), *Pseudotsuga taxifolia* (Douglas fir bark), *Picea abies* (European spruce), *Chamaecyparis obtuse* (blunt-leaved cypress), *Pinus silvestris*, *Pinus roxburghii*, *Pinus sibirica*, *Cedrus deodara*, *Juniperus communis,* and *Taxus chinensis* [3,12,13,14,15,16,17,18]. In addition, it is revealed in *Carduus marianus* L. (milk thistle), *Allium sepa* (onion), *Smilax china*, *Vitex trifolia,* and citrus fruits [9,12,19,20]. Among the plants, the larch species are usually used for isolating the compound for industry. Industrial production of DHQ is performed using water-ethanol extraction followed by chromatographic purification [8]. Recently, the *Ziziphus jujuba* seeds were found to be a new source of DHQ [21], and 470.8 µg/g of the polyphenol was obtained using ultrasonic extraction, being 4.8-fold more than that in onion. The jujube seed oil can be used as food and a pharmaceutical additive containing DHQ and silibinin.

The content of DHQ in plants does not exceed 3% [22]. In recent years, the *Saccharomyces cerevisiae* and *Yarrowia lipolytica* yeasts began to be used as a biosynthetic platform for the production of the polyphenol [23,24,25,26,27,28,29]. The highest de novo microbial titer of 4.2 g/L of DHQ was obtained by He et al. [27] in an engineered *Y. lipolytica*.

### 2.2. Chemical Structure

The DHQ molecule consists of five hydroxyl groups in the 3-, 3′-, 4′-, 5-, and 7-positions (Figure 1). It is reported that the more the number of hydroxyls linked to the aromatic ring, the higher the antioxidant activity of the polyphenol molecule [30,31].

It is assumed that the 3′- and 4′-hydroxyl groups of DHQ can participate in the inhibition of amyloid β-protein aggregation [9]. In silico stability studies by Moura et al. [32] revealed that the DHQ molecule can be susceptible to nucleophilic attack in C2′, C4, and C7 positions. The assumption was confirmed by in vitro experiments under alkaline conditions. Importantly, the hydroxylation of C5, C7, C3′, and C4′ in the flavonoid molecule was shown to increase inhibition of bacterial growth [33].

DHQ is the yellow crystalline powder that dissolves well in water–alcohol solutions and polar solvents. It can be identified as two stereoisomers of *cis*- and *trans*-forms [34]. Under the acidic and alkaline conditions, the compound exhibits UV-absorption maxima of 230 and 290 nm for *cis*- and 327 nm for *trans*-forms, respectively. Upon the pH shift from 4.5 to 8.0, the *cis*-form transforms into the *trans*-form. The (+) *trans*-DHQ form is capable of being oxidized to give quercetin, 2-(3,4-dihydroxyphenyl)-3,5,7-trihydroxy-4H-chromen-4-one. Quantitative and reliable analysis of the DHQ stereoisomers is necessary for the development of safe and effective drugs and dietary supplements containing this compound.

DHQ differs from quercetin only by the absence of a 2,3 double bond in the C ring. The compound is classified as an antioxidant because it satisfies two of the three criteria determining the ability to effectively scavenge the radicals:

(1) The presence of an o-dihydroxyl structure in the B ring provides stability;
(2) 5- and 7-OH groups with a 4-oxo function in the A and C rings are responsible for the maximum radical scavenging potential [35].

The lack of the double bond makes DHQ less effective compared to some other flavonoid antioxidants containing a 2,3 double bond. Upon interaction of DHQ with glyoxylic acid, the condensation led to the formation of the polyphenol dimer linked by a carboxymethine bridge at the C-6 and C-8 positions of the A ring [36]. The luminol-dependent chemiluminescence method revealed that the double molecule possessed higher ROS-scavenging and metal-binding activities than the initial DHQ.

Complexes of DHQ with different compounds have become widespread. Among them, it is worth mentioning the DHQ/arabinogalactan complex [37]. It provides a high antioxidant status and immunomodulatory effect due to dispersion, electrostatic, and non-covalent interactions of molecules. Inter- and intramolecular H-bonds participate in the functioning DHQ/arabinogalactan complex [37]. In addition, glycosides of DHQ isolated from different plants or biosynthesized demonstrated some pharmaceutical benefits [38,39]. Biotechnological production of taxifolin and its glycosylated, acetylated, methylated, and sulfated derivatives has developed recently [40,41]. The hydroxyl groups of DHQ, especially the 3-hydroxyl and 7-hydroxyl, are primarily responsible for forming these complexes [41]. The glycosylation of flavonoids results in higher solubility and stability, as well as bioavailability.

### 2.3. Solubility

DHQ is poorly water-soluble at room temperature (0.87–1.00 mg/mL at 25 °C) [42] with a slow rate of dissolution and absorption. It can reduce its effect in the treatment of various diseases. Different methods were performed to increase the dissolution kinetics of DHQ. Karlina et al. [43] created a microemulsion consisting of 2% DHQ, surfactant Tween 80, cosurfactant propyleneglycol, oil component Labrafil M 1944 CS, and water. The authors revealed in vitro uniform prolonged release of DHQ from the microemulsion.

To overcome the low water solubility of the compound DHQ and its derivatives, penta-O-acetylsalicylate dihydroquercetin, penta-O-acetate dihydroquercetin, and penta-O-benzoate dihydroquercetin were prepared in liposomal form and in the form of fat emulsions [44]. Moreover, to enhance the water solubility, aminomethylated derivative of DHQ was synthesized [45]. It was shown that the solubility and dissolution rates of aminomethylated DHQ were 16.3- and 6.3-fold higher than those of DHQ, respectively.

Zinchenko et al. [46] inserted DHQ into the ring of β-cyclodextrin to increase its water solubility. The obtained complex was unstable, but it provided slow release of DHQ in the water, leading to the long-term presence of free DHQ molecules in the blood of rats. In the study of Zu et al. [47], a complex of DHQ with γ-cyclodextrin prepared after emulsion solvent evaporation in combination with lyophilization demonstrated an 18.5–19.8-fold increase in solubility, a 2.8-fold augmentation of dissolution rate, and higher antioxidant capacity compared to that of the initial DHQ. Rogovskii et al. [48] obtained inclusion complexes of DHQ and its aminomethylated derivative into a cyclodextrin shell with a higher water solubility and demonstrated their antioxidant and anti-inflammatory activities comparable with those of DHQ tested on the model of carrageenan-induced paw edema in mice.

Selivanova et al. [49] used crystal engineering for obtaining DHQ co-crystals with benzaldehyde, vanillin, cinnamaldehyde, urea, and nicotinic acid. The obtained supramolecular complexes of DHQ with some coformers showed modified solubility. The crystal engineering method leads to the self-assembly of microtubes from cylindrical crystalline nanoparticles having 100–1000-fold higher solubility of DHQ compared to that in the original crystalline substance [8]. Terekhov et al. [50] performed “green” synthesis of the DHQ lyophilizates based on the mixtures of water with ethanol or acetonitrile. The obtained new phase modifications of DHQ were presented as amorphous nanostructured materials with enhanced water solubility. It is important to emphasize that upon lyophilization, neither the molecular structure of the compound nor its antioxidant properties changed. Modification of DHQ in the form of spheres via spray drying allowed Taldaev et al. [51] to enhance its water solubility by 2.225 times at room temperature. The authors supposed that the DHQ spheres can be used in the form of orally dispersed tablets with a delayed release.

The release of DHQ depends on the components it is associated with, as well as on the method of application. Hassan et al. [52] fabricated a membrane on the basis of chitosan and hyaluronan loaded with phosphatidylcholine dihydroquercetin. Upon swelling, the formed polyelectrolyte network increased the pore size, and the release of phosphatidylcholine dihydroquercetin was facilitated. The dressing based on the membrane has a promising potential for the promotion of wound healing.

### 2.4. Toxicity

DHQ is characterized by a favorable safety profile [51,53]. Nevertheless, an assessment of the effect of various carriers containing DHQ on a human body is necessary in each specific case [54,55]. After multiple-dose administration, the possibility of a cumulative action of the flavonoid should be taken into account [56]. In addition, pharmacokinetic parameters of DHQ may change depending on the way of administration of polyphenol into the body. Taldaev et al. [51] used spray drying to fabricate the DHQ spheres. The structures were amorphous in nature and stable under standard conditions, though they were not associated with a polymer carrier. The obtained DHQ spheres were tested for toxicity using the Madin–Darby canine kidney cells and assigned to Class 1 of the Biopharmaceutical Classification System.

The acceptable daily intake for the compound is expressed in mg/kg body weight per day and is 15 [8,9]. At the same time, biotransformation of the compound occurs quite quickly [8,18].

## 3. Bioavailability of DHQ

Different methods of nanotechnology were intensively developed to increase the bioavailability of DHQ (Table 1). Zu et al. [57] prepared DHQ nanoparticles with liquid antisolvent precipitation (LAP) using ethanol as the solvent and water as the antisolvent. The solubility and the dissolution rate of DHQ nanoparticles obtained under the optimal LAP conditions proved to be about 1.7- and 3.0-fold more compared to those for initial DHQ, respectively, taking into account the type of surfactant, DHQ concentration, volume ratio of antisolvent to solvent, precipitation temperature, stirring, time, and dropping speed. Moreover, their bioavailability was enhanced 7-fold more than that of the initial DHQ determined in blood samples taken from female Sprague-Dawley rats.

The antioxidant capacity of the structures also increased. Li et al. [58] applied the antisolvent method to create zein-caseinate nanoparticles loaded with DHQ. In the study, zein nanoparticles were used as the oral delivery carrier for DHQ, and sodium caseinate served as the stabilizer of the surface coating for nanoparticles. Encapsulation of flavonoid in the nanoparticles reached 80%. The obtained nanoparticles increased the DHQ concentration in rat plasma by 1.48 times, and the absolute bioavailability of polyphenol was enhanced from 0.35 to 0.52%. Sundraraman et al. [59] fabricated polyethylene glycol-coated zinc oxide nanoparticles encapsulated with DHQ. The nanoparticles were able to release DHQ depending on pH and initiate apoptosis in the human breast cancer cells.

It has been demonstrated that DHQ, interacting with iron ions in the presence of polyvinylpirrolidone (PVP), formed nanoenzymes with high biocompatibility in vitro and in vivo [60]. The nanoenzymes demonstrated the activities of catalase and superoxide dismutase under gastrointestinal conditions. Fe-DHQ nanoenzymes decreased inflammation, oxidative damage, and cell apoptosis by regulating the nuclear factor-erythroid 2-related factor 2 (Nrf2), NF-κB, Bax/Bcl-2, and VEGF signaling pathways and the levels of reactive nitrogen and oxygen species. In another study [61], DHQ-Cu-CS-nanozyme-based coating films having high free radical scavenging and peroxidase-like activities and glutathione reduction ability were prepared. The obtained structures were successfully applied for food preservation against bacterial spoilage.

Solid nanodispersion of dihydroquercetin dihydrate in the presence of PVP was obtained [62]. Upon co-precipitation of DHQ with PVP at a ratio of 1:10, scanning electron microscopy revealed round and smooth nanoparticles of 150 nm in size. The in vitro kinetics of the compound release from the nanodispersion exceeded that for raw DHQ or a physical mixture of DHQ and PVP, which can be explained by a simultaneous decrease in particle size, loss of crystallinity, and increased wettability due to the presence of a hydrophilic polymer. Yang et al. [18] used nanodispersion of DHQ-PVP prepared using the co-precipitation method and physical mixture of the same reagents (DHQ:PVP = 1:10) for intravenous and oral administration in rats. Pharmacokinetics using the UPLC-MS/MS technique revealed that the absolute bioavailability of DHQ was determined as 0.75% for DHQ-PVP nanodispersion and 0.49% for DHQ, respectively. Thus, the nanodispersion has noticeably improved the oral bioavailability of the DHQ due to enhancing its content in rat plasma.

Another approach to increase the DHQ bioavailability was proposed by Zhang et al. [63]. The authors created a nanocomposite membrane containing chitosan (CS), PVP, and DHQ, which was effective in the skin wound healing in mice due to the induction of the autophagy pathway and enhanced levels of pan-keratin, vascular endothelial growth factor VEGF and CD31. Furthermore, the membrane of the same composition protected UV-damaged human skin keratinocytes, blocking oxidative stress, inflammation, and apoptosis induced by the UV-radiation-induced MAPK/Nrf2 signaling pathway.

In another study [64], core–shell nanofiber membranes with improved antibacterial, antioxidant, and cytocompatible properties were fabricated using coaxial electrospinning utilizing polycaprolactone and silk fibroin as the Janus shell and DHQ and silk fibroin as the core. The release of DHQ continued for 28 days according to first-order release kinetics.

Abou-Taleb et al. [19] loaded DHQ in the chitosan nanoparticles (DHQ-CS NPs) and then included them in a mucoadhesive thermosensitive gel. The obtained DHQ-CS NPs gel had a greater release rate of DHQ compared to the DHQ gel. The intranasal DHQ-CS NPs gel is a promising formulation to cure Alzheimer’s disease.

Wang et al. [65] found that the sodium alginate (SA)/poly(vinyl alcohol)(PVA)/dihydroquercetin nanofibers accelerated diabetic wound healing through modulating the inflammation reaction, angiogenesis, and the level of microorganisms in skin. The obtained SA/PVA/DHQ nanofibers had high thermal stability, hydrophilicity, and improved mechanical properties. The determined cumulative DHQ release rate was 64.6 ± 3.7% at 24 h. In vitro release data demonstrated that the resulting SA/PVA/DHQ is a rapid-release complex, typically delivering the drug within a few hours or one to two days. The 16S rRNA sequencing data demonstrated that SA/PVA/DHQ optimized the wound surface microbial diversity and the skin microbiota content in diabetic rats.

Liposomes incorporated with DHQ are capable of penetrating the cells by endocytosis and increasing the therapeutic effect of the drug. Application of the DHQ-liposome dressing in burn wounds decreased the process of secondary necrosis and increased the skin reparation [66]. Encapsulation of DHQ in liposome nanoparticles (DHQ-L) enhanced the release and bioavailability of the compound in PVA/CS/DHQ-L nanocomposite membranes prepared by electrostatic spinning [67]. The structures showed improved water absorption, water vapor transmission rate, hydrophilicity, and antioxidant and antibacterial properties. Their action resulted in inhibition of activation of the IκBα/NF-kB signaling pathway, and related pro-inflammatory factors were observed, increasing the expression of CD31 and VEGF in skin tissues in wound healing in diabetic mice. In another study [68], DHQ-L prepared by film dispersion and modified with CS demonstrated enhanced bioavailability and improved kidney injury in diabetic mice. The structures inhibited the activation of the NF-κB/NLRP3/caspase-1/IL-1β signaling pathway and diminished the kidney damage.

Special enteric coating material containing hydroxypropylmethylcellulose-acetate succinate was prepared to modify DHQ-loaded liposomes [69]. The thin-film dispersion method was used to receive these structures, and they were successfully tested in vitro and in vivo in the reparation of liver damage due to modulation of inflammation and the autophagy signaling pathway. The application of the material can improve the oral bioavailability of DHQ. In another study [70], DHQ-L was incorporated in PVA/carboxymethyl chitosan-based hydrogels created using repeated freeze-thawing to increase the DHQ bioactivity. The authors successfully tested this nanomaterial using a diabetic mouse trauma model. Ding et al. [71], to achieve slow DHQ release in long-term treatment, modified DHQ-L with thiolated chitosan (CS-SH) and loaded them into a hydrogel containing carboxymethyl chitosan and oxidized dextran. This hydrogel was effective in stimulating osteoblast proliferation in rats due to the regulation of the Wnt signaling pathway. In addition, it demonstrated high antioxidant activity in vitro and the *Escherichia coli* and *Staphylococcus aureus* growth suppression with inhibition rates of 94 and 89%, respectively.

DHQ-encapsulated vesicular systems (liposomes, niosomes, and transfersomes supplemented with soybean lecithin, Tween 80, Span 60, and cholesterol) were prepared using the ethanol injection method [42]. Transfersomes demonstrated the best DHQ-loading efficacy (72–75%) and a high antioxidant activity. Authors found that in vitro release of DHQ from nanovesicles depends greatly on pH and does not exceed more than 10% under alkaline conditions mimicking the intestines, but it is more than 90% under the acidic conditions mimicking the stomach. The resulting transfersomes can be used as a promising food additive and health care product.

Jiang et al. [72] designed a drug delivery system containing hollow mesoporous whitlockite nanoparticles loaded with DHQ and encapsulated into gelatin methacryloyl hydrogel. This system was successfully applied in the treatment of osteoporotic cranial defects of ovariectomized rats.

Thus, the listed studies indicate that diverse nanomaterials have been developed in which the interaction of different organic molecules (γ-cyclodextrin, stearic acid, PVP, CS, PVA, SA, and others) with DHQ leads to an improvement in the availability of flavonoid.

Improving the solubility and bioavailability of DHQ is crucial when using it as a drug. It is important to use an effective method of delivering the polyphenol to target cells. One of the techniques is the application of a hydrogel having a large volume of water in its swollen state, without dissolving, and providing a gradual release of DHQ. Kundrapu et al. [55] encapsulated DHQ in the self-assembled bovine serum albumin (BSA) hydrogel to treat triple-negative breast cancer cells. DHQ-BSA hydrogel demonstrated 90% entrapment efficiency and 96% release of DHQ in the in vitro experiments with the MDA-MB 231 and MDA-MB 468 TNBC cell lines. Encapsulation of DHQ into BSA hydrogel significantly increased its cytotoxicity against breast cancer cells.

When using DHQ as a food additive, it should be taken into account that the bioaccessibility of DHQ, like other polyphenols, may be limited by its interaction with various carbohydrates, proteins, and other compounds, which are part of food components [73].

The decrease in bioavailability caused by a low solubility is not unique to DHQ but also exists for other polyphenols. Recently, some approaches to solving the problem have been developed [74]. Using conjugation methods based on chemical and physical grafting of polyphenols onto edible polysaccharides, certain progress has been achieved in increasing the bioavailability of the compounds included in health-beneficial food [74].

**Table 1:** The developed nanomaterials containing DHQ.

The Nanomaterial Type	The Composition of Nanomaterial	Experiments		Possible Application	Reference
Nanoenzymes	DHQ-Fe in the presence of PVP (the activities of catalase and superoxide dismutase)	in vitro	in vivo	Ethanol-induced gastric ulcer treatment	[60]
	DHQ-Cu-CS-nanozyme-based coatings	in vitro		Fruit preservation	[61]
Nanosuspension	DHQ-γ-cyclodextrin	in vitro	in vivo	New oral DHQ formulation	[47]
Nanoparticles	DHQ-PVP	in vitro		New oral DHQ formulation treatment	[62]
	DHQ prepared by liquid antisolvent precipitation	in vivo		New oral DHQ formulation	[57]
	DHQ encapsulated in zein-caseinate	in vivo		New oral DHQ formulation	[58]
	DHQ-loaded in polyethylene glycol-coated zinc oxide	in vivo		Cancer therapy	[59]
Nanocomposite membrane	CS-PVP-DHQ	in vivo		Treatment of UVA-induced skin injury	[63]
Core–shell nanofiber membrane	Polycaprolactone and silk fibroin	in vitro		Antibacterial and antioxidant agent	[64]
Liposome nanoparticles	hydroxypropylmethylcellulose-acetate succinate-DHQ-L	in vitro	in vivo	Reparation of liver injury	[69]
	DHQ-L-CS				
Nanocomposite membrane with liposome nanoparticles	PVA/CS/DHQ-L	in vitro	in vivo	Diabetic wound treatment	[67]
Nanovesicles	Transfersomes supplemented with lecithin and Tween 80 in the presence and absence of cholesterol	in vitro		Delivery of DHQ to food ingredients	[42]
Gel containing nanoparticles	DHQ-CS NPs-mucoadhesive thermosensitive gel	in vitro	in vivo	Amelioration of Alzheimer disease	[19]
	Mesoporous whitlockite nanoparticles loaded with DHQ and encapsulated into gelatin methacryloyl hydrogel	in vitro	in vivo	Reparation of osteoporosis	[72]
Gel containing liposome nanoparticles	PVA-carboxymethyl chitosan hydrogel with DHQ	in vitro	in vivo	Diabetic wound healing	[70]
	DHQ-L modified with thiolated chitosan and loaded into hydrogel containing carboxymethyl chitosan and oxidized dextran	in vitro	in vivo	Reparation of skull defects	[71]
Nanofibers	SA-PVA-DHQ	in vitro	in vivo	Diabetic wound healing	[65]
Amorphous nanostructured material	DHQ lyophilizates based on mixtures of water with ethanol or acetonitrile.	-		Pharmaceutical ingredient for injectable dosage form	[50]

Abbreviations: DHQ—dihydroquercetin; PVP—polyvinylpirrolidone; CS—chitosan; L—liposomes; PVA—polyvinyl alcohol; NPs—nanoparticles; SA—sodium alginate.

The methods that were used to verify that the new nanostructures with DHQ increased their solubility include dissolution testing. The solubility of the DHQ-loaded nanostructure is compared to that of the initial unprocessed DHQ. The dissolution profile of the initial DHQ serves as a critical reference point. This test can be based on the results of UV spectrometry [50] or HPLC [62]. The dissolving capability test and solvent residue test are also carried out [47]. Moreover, other methods, such as mass spectrometry [50], nuclear magnetic resonance [50], Fourier-transform infrared spectroscopy (FTIR) [47,50,57,59,65,69,72], X-ray diffraction (XRD) [47,57,59,62,65,72], scanning electron microscopy [47,57,59,65,70,72], Raman spectroscopy [62], and ultraHPLC with quadrupole time-of-flight mass spectrometry [58], are used too. They present the different characteristics of the prepared nanostructures and confirm the creation of the new phase, which is indirectly linked to improved solubility of DHQ.

This review part shows that researchers are attempting to apply the DHQ-nanoderivatives in various fields of medicine and the food industry. However, it is important to remember that nanostructures are characterized by chemical and physical instability. At the same time, an increase in the production of DHQ-nanoderivatives from the laboratory to industrial scale is associated with a number of difficulties and can be expensive. To stabilize these structures, it seems promising to use biodegradable polymers (which is already being performed using chitosan). In addition, different encapsulation methods can help protect the DHQ from fast metabolism until it reaches the target site. Moreover, when using nanomaterials containing DHQ, it is important to control the type of stereoisomer of flavonoid in the final product.

Many developed nanomaterials are promising new oral DHQ formulations and dietary supplements, but they must be thoroughly tested for toxicity before use. The safety of their higher doses or long-term supplementation is not well-documented. Assessing the safety of nanomaterials containing DHQ is an important and complicated goal, since, in addition to the possible toxic effects of DHQ, the nanostructures themselves can have a harmful effect. The most commonly used method for determining the toxicity of DHQ-containing nanoderivatives is the 3-(4,5-dimethylthiazol-2-yl)-2,5-diphenyltetrazolium bromide (MTT) assay. The MTT method was applied for the detection of toxicity of Cu DHQ/CS nanozymes in human gastric epithelial cells GES-1 [61], DHQ composite nanofibrous membranes [63], and SA/PVA/DHQ in human keratinocyte HaCaT cells [65]. Moreover, the toxicity of Cu DHQ/CS nanozymes was detected by a hemolysis test on red blood cells [61].

Among the various DHQ-delivery systems, nanostructured lipid carriers appear to be the most promising, as they are expected to retain a significant drug payload in the human body. These systems use nanotechnology to encapsulate and protect DHQ, enhancing its therapeutic potential.

## 4. Antibacterial Action of DHQ

DHQ, like other plant polyphenols, is of interest as a possible alternative to antibiotics. The in vitro comparison of antimicrobial action of different antibiotics (tetracycline, chloramphenicol, streptomycin, bacitracin, grisin, benzylpenicillin at 3.0, 5.0, 10.0, 16.0, 19.0, 24.0, and 48.0 µg/mL each) and 0.5, 1.0, 2.0, and 5.0% DHQ against *Staphylococcus epidermidis*, *Micrococcus lysodeicticus*, *E. coli*, and *Pseudomonas aeruginosa* was performed by Artem’eva et al. [75]. Based on the gel diffusion method, it was found that *S. epidermidis* had high sensitivity to 5.0% DHQ but low susceptibility to bacitracin at all the concentrations tested and to grisin in the range of 3–10 µg/mL. On the other hand, *E. coli* and *M. lysodeicticus* showed low sensitivity to 0.5–2.0% DHQ, but the bacteria had high susceptibility to all the antibiotics tested. Based on the results that the flavonoid inhibited the growth of facultative pathogens but did not significantly affect probiotic bacteria, the authors suggested using DHQ as an alternative to antibiotics.

Yang et al. [76] studied the action of DHQ against foodborne bacteria, namely *E. coli* and *S. aureus*, and determined that their MIC and MBC were 1.11 and 2.22 mg/mL and 0.556 and 1.11 mg/mL, respectively. The flavanonol was found to cause damage to the cell wall and membrane, change the cell morphology, reduce bacterial biofilm formation, and enhance the superoxide dismutase and alkaline phosphatase activities. DHQ, being the inhibitor of proliferation of the Gram-negative and Gram-positive bacteria, can be considered a promising agent for milk preservation.

The application of the agar disk diffusion test demonstrated that DHQ (along with some other polyphenols of resveratrol and dihydromyricetin) was effective against the growth of the Gram-positive pathogenic bacterial dermatophyte, namely *S. aureus*, even at the smallest possible concentration of 0.22 mM, which exceeds the molar activity of some antibiotics traditionally used as dermatoprotective external agents [77].

The significant interaction of DHQ with DNA gyrase and isoleucyl-tRNA synthetase in *Mycobacterium tuberculosis* was revealed using a docking study and free energy calculations [78]. In DNA gyrase, flavanonol participated in the formation of the hydrogen bond with Gly77 and hydrophobic interactions with amino acids Asn46, Arg76, Ile78, Pro79, Gly101, Lys103, and Thr165. Further experiments in vitro showed comparable MIC values against this bacterium for DHQ and standard antibiotics. The application of molecular docking in another study [79] demonstrated that DHQ was able to bind β-ketoacyl acyl carrier protein synthase III, which is a key enzyme in fatty acid biosynthesis in *Enterococcus faecalis*. It was shown that hydrogen bonds between the 5- and 4′-hydroxy groups and the side chain of Arg38 and the backbone carbonyl of Phe308 are the main points for interactions inhibiting the enzyme in *E. faecalis* and its vancomycin-resistant isolates. 

Oh et al. [80] revealed synergistic activity of DHQ with antibiotics against different *Campilobacter jejuni* strains, namely, decreasing MICs of ciprofloxacin and erythromycin by 32- and 16-fold, respectively, at a concentration of flavonoid of 8 µg/mL. Moreover, the addition of DHQ increased antibiotic accumulation in *C. jejuni* and its membrane permeability. The compound reduced the level of expression of *cmeABC*, encoding a multidrug efflux pump, providing the resistance of the bacterium to antibiotics.

DHQ is a promising agent in the fight against antibiotic-resistant bacteria, such as methicillin-resistant *S. aureus* (MRSA) [81]. It was shown that flavanonol is able to reversibly inhibit the activity of the cysteine transpeptidase sortase A of MRSA. The enzyme participates in binding surface proteins to the cell wall and provides biofilm formation during pathogen attack [82]. An addition of DHQ decreased the adhesion of *S. aureus* in vitro and the biofilm formation. The main amino acid residues of the enzyme that bind DHQ were Asp-170 and Gln-172. In vivo experiments demonstrated that intranasal administration of DHQ every 12 h protected mice from pneumonia caused by lethal doses of *S. aureus*. In addition, Abid et al. [83] reported antibacterial and antibiofilm activities of DHQ against vancomycin-resistant *S. aureus* (VRSA).

Kuspradini et al. [84] showed that DHQ and flavanonol rhamnoside isomers isolated from a 50% aqueous ethanol extract of *Koompassia malaccensis* caused 90% inhibition of the growth of the major dental caries bacterium *Streptococcus sorbinus* and 80% inhibition of its glucosyltransferase activity. Wang et al. [85] revealed the inhibiting action of DHQ in vitro with an MIC of 1 mg/mL against the Gram-positive facultative caryogenic anaerobe *Streptococcus mutans* and found the destruction of biofilm formation and prevention of the tooth enamel demineralization caused by the bacterium. Moreover, the authors confirmed the efficacy of DHQ against dental caries and its safety in vivo using the rat dental caries model.

The protective effect of DHQ against another type of disease, namely dextran sulfate sodium (DSS)-induced colitis, was also observed [86]. The normalization of the gut bacterial microflora and the decrease in Clostridium spp., resulting in a reduction in the severity of colitis, were revealed in this case. Li et al. [87] found that the supplement of DHQ in mice with DSS-induced intestinal mucositis decreased the weight loss and diarrhea in animals. Furthermore, it altered the ratio of different bacteria in the gut microbiome, specifically increasing the relative abundance of *Akkermansia* and *Lactobacillus* while decreasing the abundance of Bacteroides. This shift led to an increase in the production of butyric and isobutyric acids in the feces. Gelatinized-retrograded corn starch was used for delivering DHQ in mice with colitis induced by DSS [88]. The treatment by DHQ improved intestinal flora diversity, reducing the number of inflammation-related bacteria and increasing that of beneficial bacteria like Lachnospiraceae. In another study, DHQ decreased the *Prevotella copri*-caused intestinal injury and the reduction in growth in weaned piglets by modulating the composition of gut microbiota and bile acids [89].

DHQ-loaded gastroadhesive microparticles (DHQ-MP) developed by Moura et al. [90] were effective in vitro against *Helicobacter pilori*, a Gram-negative bacterium causing numerous stomach diseases. The MIC value of DHQ for *H. pilori* was 625 μg/mL. Moreover, the oral treatment with DHQ-MP (81.37 mg/kg, containing 12.29% of DHQ) twice a day for 7 days or DHQ alone (10 mg/kg) reduced the ulcer area by 63 or 40%, respectively, compared to the control untreated rats. Moreover, the authors found a reversible interaction of DHQ with H^+^/K^+^-ATPase in silico.

The results of this part are presented in Table 2.

## 5. Antiviral Action of DHQ

Many flavonols provide antiviral action [91,92,93,94]. Among the viral diseases, which are sensitive to the action of DHQ and have been studied in detail recently, we can highlight pathologies caused by enteroviruses, coronaviruses, and retroviruses.

Galochkina et al. [95] found that DHQ effectively inhibited coxsackie virus B4, belonging to the genus *Enterovirus*, in the mice model of viral pancreatitis. The compound (75 or 150 mg/kg once a day for five days) caused a dose-dependent decrease in the virus titer in pancreatic tissue upon intraperitoneal injection. In addition, an increase in antioxidant levels and a decrease in inflammation were observed compared to those in the untreated control. The authors suggested that the high antiviral activity of DHQ could be explained by three possible mechanisms, namely direct antiviral action against the definite, still unidentified viral protein; alteration of ROS-mediated signals or metabolic reactions required for viral replication; and reduction in ROS levels in inflammatory tissues, thereby reducing tissue damage. Taking into account that DHQ is more effective than the reference compound of ribavirin, it is possible to conclude that the flavanonol is a promising drug in the complex treatment of virus-induced pancreatitis.

Upon screening the library containing over 606 million compounds, DHQ was identified as a potential inhibitor of the main protease (Mpro) of SARS-CoV-2, the coronavirus causing COVID-19 [96]. Mpro is considered to be essential for viral replication. Among 44 citrus flavonoids, DHQ has the lowest predicted IC_50_, confirming its potential role as an inhibitor of Mpro [97]. Al-Karmalawy et al. [98] calculated its IC_50_ as 78.69 mg/mL. Based on docking simulation results, Zhu et al. [92] found that some flavonols and dihydroflavanols, including DHQ, were capable of inhibiting the activity of SARS-CoV-2 Mpro. The authors found that the compounds could bind to at least two subsites (S1, S1′, S2, and S4) in the binding enzyme pocket and inhibit the activity of the virus. Moreover, dose–response curves obtained in vitro demonstrated that at the concentrations above 2.5 μM, DHQ and isoquercitrin inhibited the replication of HCoV 229E, causing the common cold. The above compounds can be used in the design of new drugs and medication regimens for the treatment of COVID and colds. Orlova et al. [8] emphasized that in the COVID therapy, it is essential to consider the route of administration (intravenous or peroral) and bioavailability of the dosage form, absorption time, elimination half-life, and the influence of other components in combination treatment. The clinical trial evaluating an application of DHQ as a post-COVID-19 therapy supplement was performed [91].

The combination therapy is used to improve the quality of life of patients with acquired immunodeficiency syndrome caused by human immunodeficiency virus (HIV). One of its promising components is DHQ, isolated from the barks and roots of *Cassia abbreviata* and having anti-HIV activity with an IC_50_ of 49.04 µM [99]. On the other hand, Zheng et al. [100] revealed that purified DHQ obtained from the tropical tree prevented HIV-1 entry in different types of human cells at an IC_50_ of 240 µM.

The results of this part are presented in Table 3.

## 6. Anti-Age Effect of DHQ

Recently, in many countries, there are more and more aging people, and new drugs are needed to improve the expectancy and quality of life and to provide protection against age-related diseases. The study of the participation of DHQ in the aging process is at the start. It is well known that defense systems against oxidative stress and inflammation get worse with age, and DHQ, as a powerful antioxidant, attracts the attention of researchers. To test the anti-aging effect of the flavonoid, different eukaryotic organisms were under investigation.

Polyextremophilic non-pathogenic ascomycetous yeast *Y. lipolytica* can serve as a model to study aging processes. The cells are able to survive at extreme pH (from 2.5 to 9.5) and temperatures (up to 38 °C) and in the presence of toxic compounds due to their ability to process reactive oxygen species (ROS). The molecules can cause oxidative damage to DNA, proteins, and membrane lipids, being the reason for many pathologies, including age-related ones [101]. In the cell, the main process of ROS formation occurs in the mitochondria; therefore, the structures are key participants in the aging process. However, it should be taken into account that moderate ROS production activates “mitohormesis”, an adaptive response to moderate stress, increasing the lifespan of the cell [102]. The action of DHQ was evaluated in the *Y. lipolytica* cells serving as a model to study the survival ability upon long-lasting cultivation [103]. Under these conditions, chronological aging of the cells was detected, characterized by an increase in ROS levels accompanied by a decrease in cell number, size, colony-forming units, respiratory rate, and membrane potential. At the 6-week-old growth stage, the supplement of 150 μM DHQ diminished the ROS formation, but the effect was more significant when it was administered with the oxidant 2,2′-azobis (2-amidinopropane) dihydrochloride (AAPH) compared to the control samples of the same age. It can be supposed that AAPH increases the antiradical protection of DHQ, inhibiting the chain reactions of lipid peroxidation. Being a powerful antioxidant, DHQ is able to have a protective effect on oxidative-reductive processes in the cell. The action was shown to be expressed in the inhibition of superoxide anion radical production in *Y. lipolytica* at elevated growth temperatures [104]. Furthermore, polyphenols can inhibit cytochrome P450 and lipid peroxidation in the mitochondria to overcome oxidative stress [35]. Upon long-term growth, DHQ caused time-dependent changes in different metabolic parameters of yeast [103]. The flavonoid declined CFU at 1–4 weeks of growth, decreased metabolic activity after 1 day of cultivation, and enhanced that on 14–56 days of growth. It induced the cell respiration rate after 24 h of growth and inhibited the alternative mitochondrial oxidase at late stationary growth stages. DHQ acted as a mild pro-oxidant in the early exponential phases and a potent antioxidant in the later phases of the *Y. lipolytica* cultivation, normalizing the cell oxidative-reductive processes, decreasing free radical oxidation in the mitochondria, and potentially extending the life expectancy of the organism.

In animals and humans, it is difficult to distinguish the effect of DHQ on the aging process and the metabolic reactions that determine the course of age-related diseases. To solve the problem, a search is underway for the models, using compounds that can artificially induce aging. D-galactose is one of the compounds. Liu et al. [105] found metabolic and histological changes in the brain tissue after induction of mouse aging by using intraperitoneal application of D-galactose (800 mg/kg once per 3 days for 12 weeks). After 6 weeks of the induction, the effect of the DHQ treatment (20 and 40 mg/kg) introduced daily by oral gavage was evaluated. The flavonoid supplement improved spatial learning and memory deterioration in mice. DHQ diminished the oxidative stress damage and apoptosis in brain tissue caused by D-galactose. It decreased the content of ROS and malondialdehyde, increased the activity of antioxidant enzymes, and modulated the activity of the phosphatidylinositol 3-kinase (PI3K)/protein kinase B (AKT) pathway, a central signaling cascade playing a crucial role in regulating cell survival. In addition, activation of Nrf2, nuclear heme oxygenase-1 (HO-1), and NADH dehydrogenase quinone 1 was observed. It is worth noting that after the DHQ administration to D-galactose-induced mice, the composition of intestinal microflora changed towards an increase in the beneficial bacteria. In turn, the predominance of beneficial microorganisms in the gut microbiota can prevent the accumulation of senescent cells, and it is able to improve overall metabolism in aged organisms [106]. The same aging model was used to study the effects of DHQ in vitro [107]. Supplement of DHQ to D-galactose-induced brain neuronal cells HT-22 enhanced the activities of superoxide dismutase and glutathione level but decreased the MDA level, protecting the cells against oxidative stress damage. It was shown that this flavonoid participates in the regulation of SIRT1/p53 and PI3K/AKT signaling pathways. Therefore, DHQ is capable of delaying the aging process caused by D-galactose in mice in vitro and in vivo.

Xie et al. [108] found that DHQ prevented the pathological processes in H_2_O_2_-induced Retinal Pigment Epithelium (RPE) cells used as a model of age-related macular degeneration. The metabolic reactions included an inhibition of the RPE cell apoptosis, intracellular production of ROS, and a cleavage of poly(ADP-ribose) polymerase. At the same time, activation of mRNA and the expression of HO-1, NAD(P)H:quinone oxidoreductase 1, and glutamate–cysteine ligase modifier were observed. The above-mentioned proteins belong to Phase II enzymes responsible for the body’s detoxification process. Moreover, it was shown that Nrf2 participated in the DHQ-induced RPE cytoprotective effect against H_2_O_2_.

## 7. Conclusions

DHQ represents a highly promising natural flavonoid with different biological effects and potential applications in medicine, pharmacy, and biotechnology. Advances in understanding its molecular mechanisms and the development of innovative delivery systems have significantly expanded the prospects for its practical use. However, limited solubility and variability in bioavailability remain key obstacles to its widespread application. To overcome the problem, multifaceted nanotechnological approaches systematized in this review were performed.

DHQ can be used as an alternative to antibiotics, is able to enhance the action of some antibiotics, and is a promising tool in the fight against a number of antibiotic-resistant bacteria. It should be noted that the compound is able to reduce the number of bacteria that cause inflammation and increase the number of bacteria beneficial to humans. Moreover, recently, DHQ has been successfully used against enteroviruses, coronaviruses, and retroviruses. Furthermore, DHQ, as a powerful antioxidant, attracts the attention of researchers due to its activity in the defense systems against oxidative stress and inflammation during aging.

Future perspectives for the DHQ development should include the following:

- Optimization of nanotechnology-based formulations to improve solubility and controlled release;
- Deeper elucidation of DHQ’s molecular targets and pathways;
- Systematic toxicological assessments in specific disease models;
- DHQ-containing medications must be protected from environmental influences such as light, oxygen, and heat to maintain their effectiveness; research is needed to develop additives in formulations that can protect DHQ from degradation;
- Quantitative and reliable analysis of the DHQ stereoisomers is necessary for the development of safe and effective drugs and dietary supplements.

Therefore, DHQ can be considered a valuable candidate for the creation of next-generation antioxidant, antimicrobial, antiviral, and geroprotective drugs.

## Figures and Tables

**Table 2:** The main items of antibacterial action of DHQ.

Item	Bacteria	Reference
Inhibition of pathogenic bacteria	*Esherichia coli* and *Staphylococccus aureus*	[76]
	*S. aureus*	[77]
	*Mycobacterium tuberculosis*	[78]
	*Enterococcus faecalis*	[79]
	*Streptococcus sorbinus*	[84]
	*Streptococcus mutans*	[85]
	*Helicobacter pilori*	[90]
Inhibition of antibiotic-resistant bacteria	Methicillin-resistant *S. aureus* (MRSA)	[81]
	*S. aureus*	[82]
	vancomycin-resistant *S. aureus* (VRSA)	[83]
Alternative to antibiotics	*Staphylococcus epidermidis*, *Micrococcus lysodeicticus*, *E. coli* and *Pseudomonas aeruginosa*	[75]
Synergistic activity with antibiotics	*Campilobacter jejuni* + ciprofloxacin and erythromycin	[80]
Normalization of the gut bacterial microflora	*Clostridium* spp.	[86]
	*Akkermansia*, *Lactobacillus,* and Bacteroides	[87]
	Lachnospiraceae	[89]
	*Prevotella copri*	[89]

**Table 3:** The main items of antiviral action of DHQ.

Virus	Possible Mechanism of Action	Reference
Coxsackie virus (enterovirus)	Direct antiviral action against the definite viral protein; alteration of ROS-mediated signals/metabolic reactions in viral replication; reduction in ROS levels in inflammatory tissues	[95]
SARS-CoV-2 (coronavirus)	Inhibition of Mpro essential for viral replication	[92,97,98]
HIV (retrovirus)	Prevention of the HIV-1 entry in different types of human cells	[100]

## Data Availability

The original contributions presented in the study are included in the article. Further inquiries can be directed to the corresponding author.
